# Spindle Cell Predominant Anaplastic Pleomorphic Xanthoastrocytoma (WHO Grade 3) With Focal Piloid Features: A Rare Case Study With Comprehensive Molecular Profiling

**DOI:** 10.7759/cureus.113858

**Published:** 2026-08-03

**Authors:** Charu Shastri, Jai Thakur, Rasha Mohammed, Osama Elkadi

**Affiliations:** 1 Department of Pathology and Laboratory Medicine, University of South Alabama Health, Mobile, USA; 2 Department of Neurosurgery, University of South Alabama Health, Mobile, USA

**Keywords:** anaplastic, braf v600e, cdkn2a deletion, high-grade astrocytoma, piloid features, pleomorphic xanthoastrocytoma

## Abstract

Pleomorphic xanthoastrocytoma (PXA) is a rare astrocytic tumor of the central nervous system. The typical form demonstrates relatively low-grade histologic features, whereas an anaplastic variant shows more aggressive behavior, including increased mitotic activity and necrotic changes. These tumors are often associated with alterations involving key growth signaling pathways and cell cycle regulatory genes, with molecular features that may resemble those seen in other high-grade astrocytic neoplasms. We describe an unusual example of an anaplastic pleomorphic xanthoastrocytoma showing focal piloid differentiation. The patient presented with acute neurologic symptoms, and imaging demonstrated a large, enhancing, well-circumscribed cerebral lesion with limited surrounding edema. Histologic evaluation revealed a highly cellular astrocytic neoplasm composed of spindle-shaped cells with marked pleomorphism, including scattered multinucleated forms, brisk mitotic activity, and necrotic areas. At the periphery, regions with elongated bipolar glial cells and occasional cytoplasmic inclusions suggestive of piloid morphology were identified. Molecular analysis demonstrated an activating alteration in the mitogen-activated protein kinase (MAPK) pathway along with additional genomic abnormalities, while mutations commonly associated with diffuse gliomas were not detected. The presence of piloid features within an otherwise anaplastic tumor is rare and may be relevant to the relatively favorable outcome observed during extended follow-up.

## Introduction

Pleomorphic xanthoastrocytoma (PXA) is a rare astrocytic neoplasm of the central nervous system (CNS), accounting for less than 1% of all astrocytic tumors [1]. Typically arising in children and young adults, PXA is characterized by its superficial location, often involving the temporal or parietal lobes, and exhibits a circumscribed growth pattern with pleomorphic xanthomatous cells, lipidized astrocytes, and eosinophilic granular bodies (EGBs) [2]. According to the World Health Organization (WHO) classification of CNS tumors, classic PXA is assigned a WHO grade 2, reflecting its relatively indolent behavior, with five-year survival rates exceeding 70%. However, anaplastic transformation to WHO grade 3 can occur, defined by the presence of increased mitotic activity (>5 mitoses per 10 high-power fields (hpf)) and/or pseudopalisading necrosis, which portends a more aggressive course and poorer prognosis. According to the 2021 WHO Classification of CNS tumors, the modifier 'anaplastic' has been discontinued, and these tumors are classified as pleomorphic xanthoastrocytoma (PXA), CNS WHO grade 3 [3].

Molecularly, PXAs are hallmarked by alterations in the mitogen-activated protein kinase (MAPK) pathway, most commonly v-Raf Murine Sarcoma Viral Oncogene Homolog B1 (BRAF) V600E mutations (up to 60-70% of cases) or less frequently BRAF-KIAA1549 fusions, alongside homozygous deletions of cyclin-dependent kinase inhibitor 2A/2B (CDKN2A) and/or CDKN2B on chromosome 9p21.3 in approximately 50-70% of anaplastic variants [2,4,5]. These features overlap with other high-grade gliomas, such as epithelioid glioblastoma (eGBM), which shares BRAF V600E and CDKN2A/B deletions but often displays more infiltrative growth and epithelioid morphology [1].

Additionally, the 2021 WHO classification introduced high-grade astrocytoma with piloid features (HGAP), a distinct entity defined primarily by its DNA methylation profile, characterized by piloid morphology (elongated, bipolar glial cells), EGBs, Rosenthal fibers, and frequent MAPK alterations without isocitrate dehydrogenase (IDH) or histone H3 mutations [3,6]. HGAP typically affects adults in the posterior fossa and exhibits aggressive behavior with frequent recurrences, though some cases show prolonged survival [7].

The coexistence of anaplastic PXA features with piloid elements is exceptionally rare, with limited reports in the literature, and raises diagnostic challenges due to morphological and molecular overlaps with HGAP and other gliomas [8,9]. Herein, we present a unique case of spindle cell predominant anaplastic PXA (WHO grade 3) with focal piloid features in a young male, accompanied by comprehensive molecular profiling that highlights these diagnostic nuances and prognostic implications.

## Case presentation

**Figure 1:**
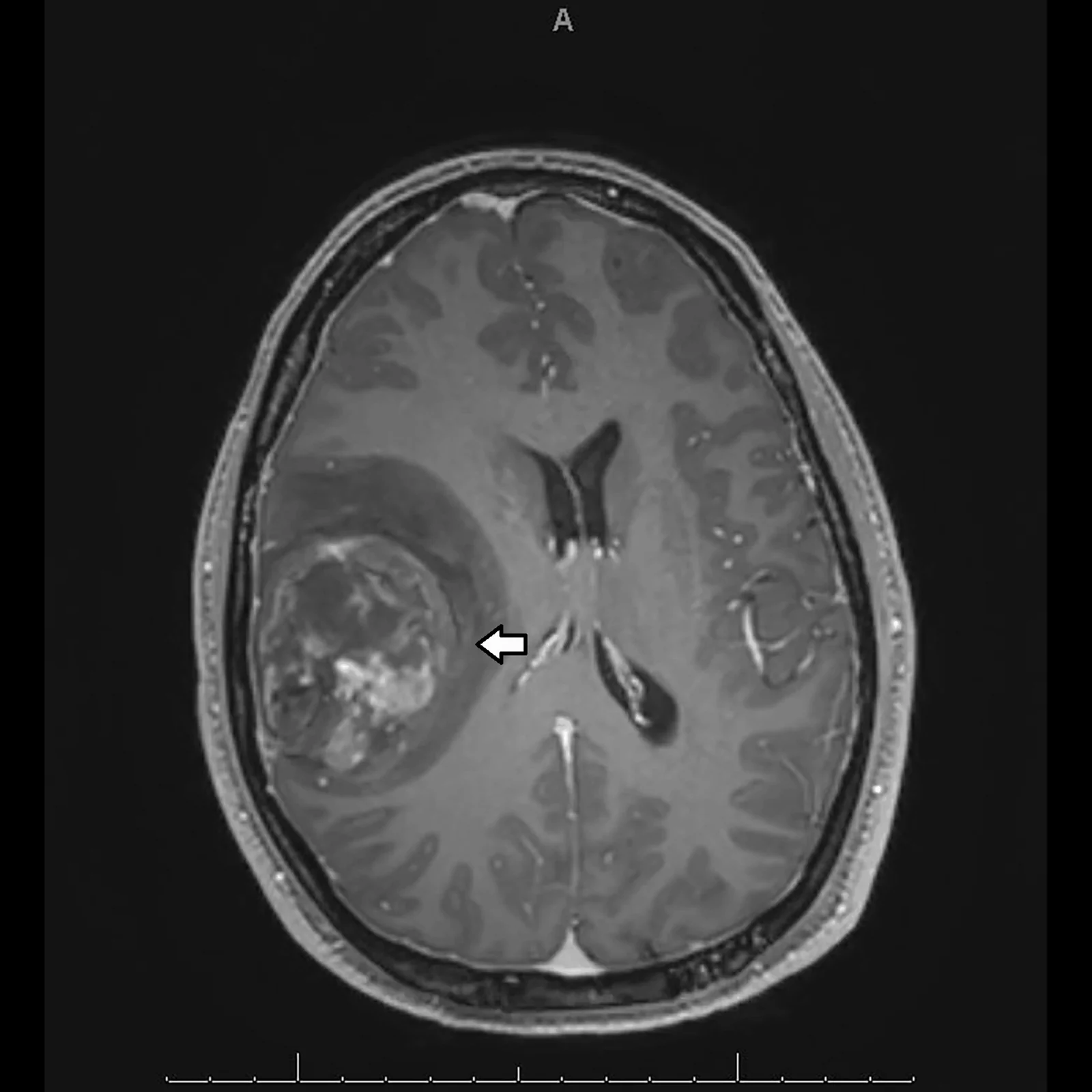
Brain magnetic resonance imaging (MRI), non-contrast, showing a tumor with peri-lesional edema. A heterogeneously enhancing left frontoparietal mass with surrounding perifocal edema measuring 6.0 x 4.5 x 5.0 cm, and mild leftward midline shift.

A 39-year-old previously healthy male presented to the emergency department with acute onset of generalized seizures, followed by expressive aphasia and a witnessed fall. Neurological examination revealed mild right-sided hemiparesis and disorientation, with no focal deficits otherwise. Brain magnetic resonance imaging (MRI) demonstrated a 6 cm heterogeneous, contrast-enhancing mass in the left frontoparietal region, exhibiting relative circumscription with mild surrounding vasogenic edema, mild midline shift, and minimal mass effect (Figure 1). The lesion involved cortical and subcortical white matter, with no evidence of hydrocephalus. Differential diagnosis included high-grade glioma, metastasis, or, less likely, primary CNS lymphoma.

The patient underwent urgent left frontoparietal craniotomy for gross total resection. Intraoperatively, the tumor was noted to be firm, well-demarcated, and superficially located, with no infiltration into deep structures. Postoperative MRI confirmed complete resection with no residual enhancement. The patient recovered well, with resolution of seizures on anticonvulsant therapy, and was discharged on postoperative day five. Adjuvant therapy included concurrent temozolomide and focal radiation (60 Gy in 30 fractions), followed by maintenance temozolomide for 12 months. Follow-up MRI at four years post-diagnosis showed no evidence of recurrence, and the patient remains in complete clinical remission. 

**Figure 2:**
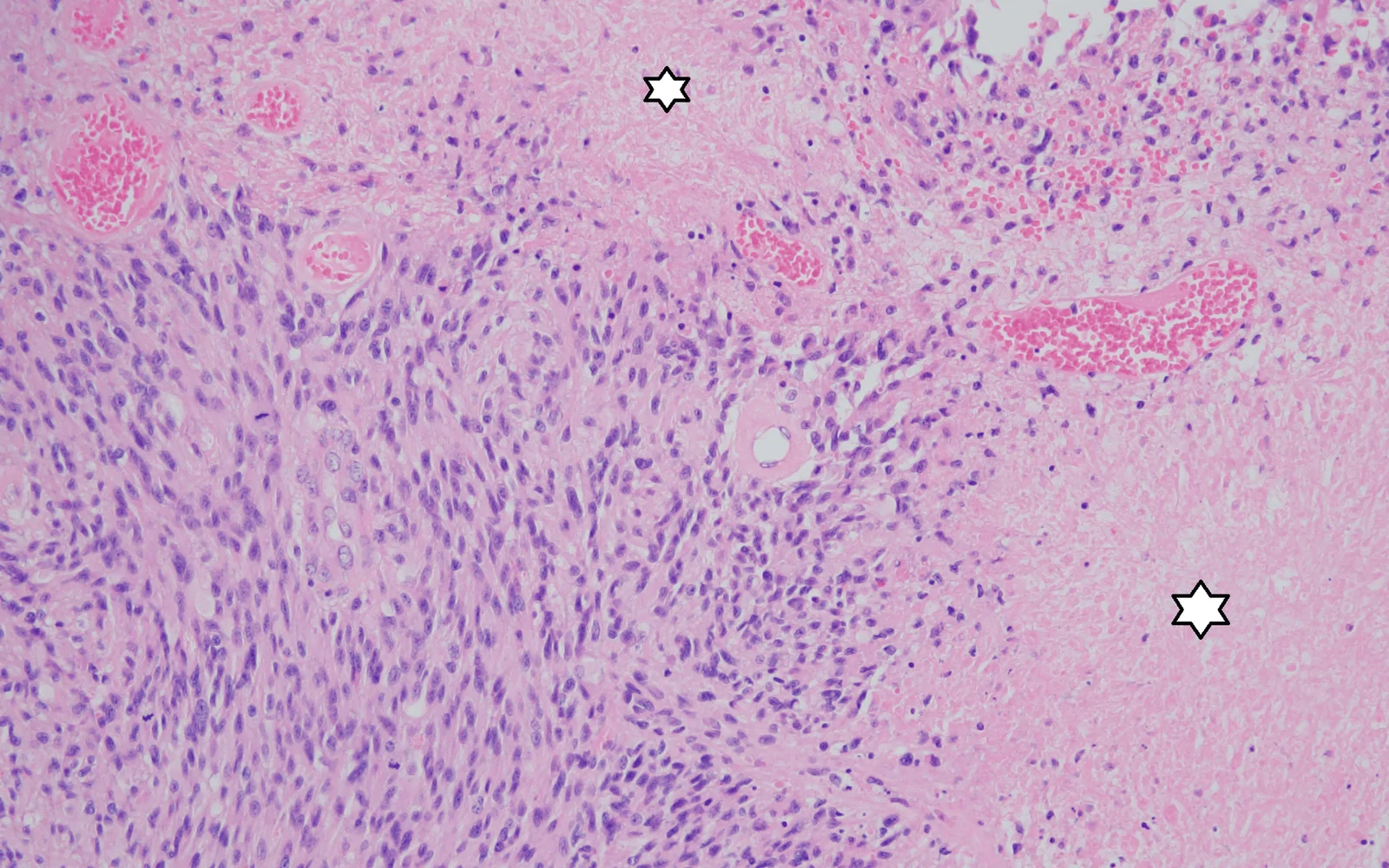
Anaplastic pleomorphic xanthoastrocytoma (PXA). Hematoxylin and eosin (H&E), low power: Tumor shows a variable degree of fascicular arrangement with palisading necrosis (indicated by a star).

**Figure 3:**
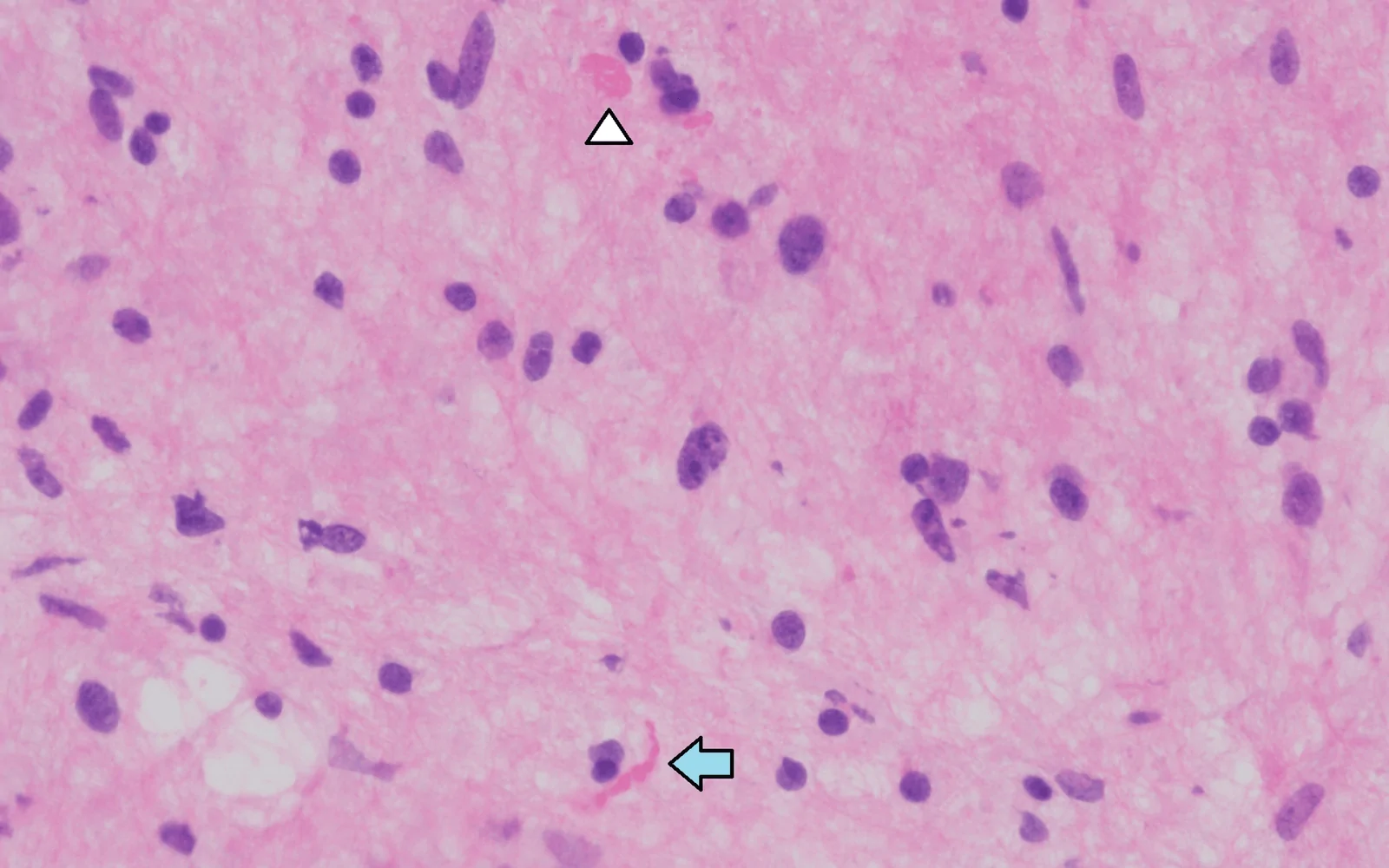
Hematoxylin and eosin (H&E): Tumor showing areas of focal piloid features with Rosenthal fibers (arrow) and eosinophilic granular bodies (arrowhead) in the background.

**Figure 4:**
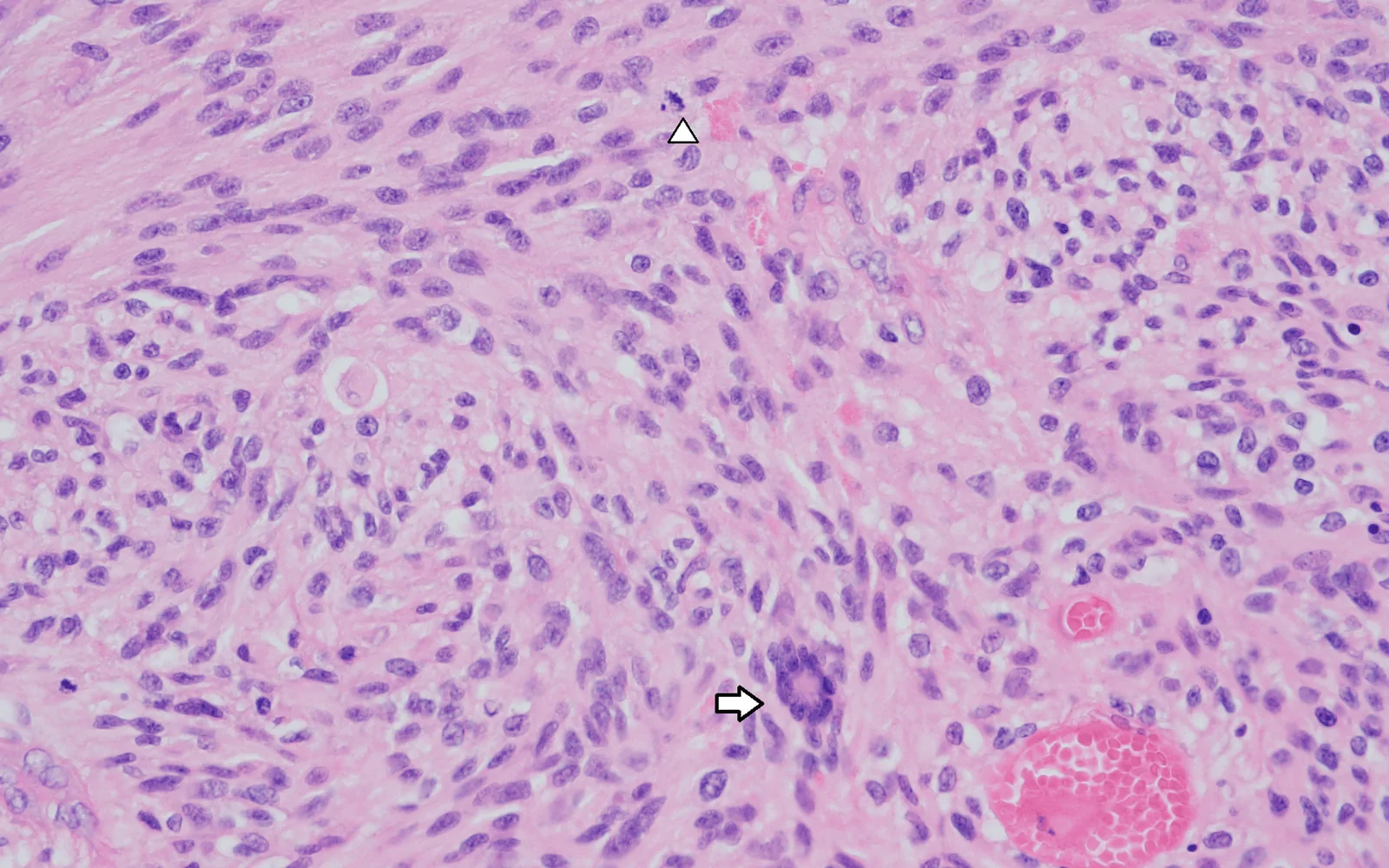
Anaplastic PXA, H&E: frequent mitoses (arrowhead) and multinucleated Touton-type giant cells (arrow) are seen. PXA: pleomorphic xanthoastrocytoma; H&E: hematoxylin and eosin.

**Figure 5:**
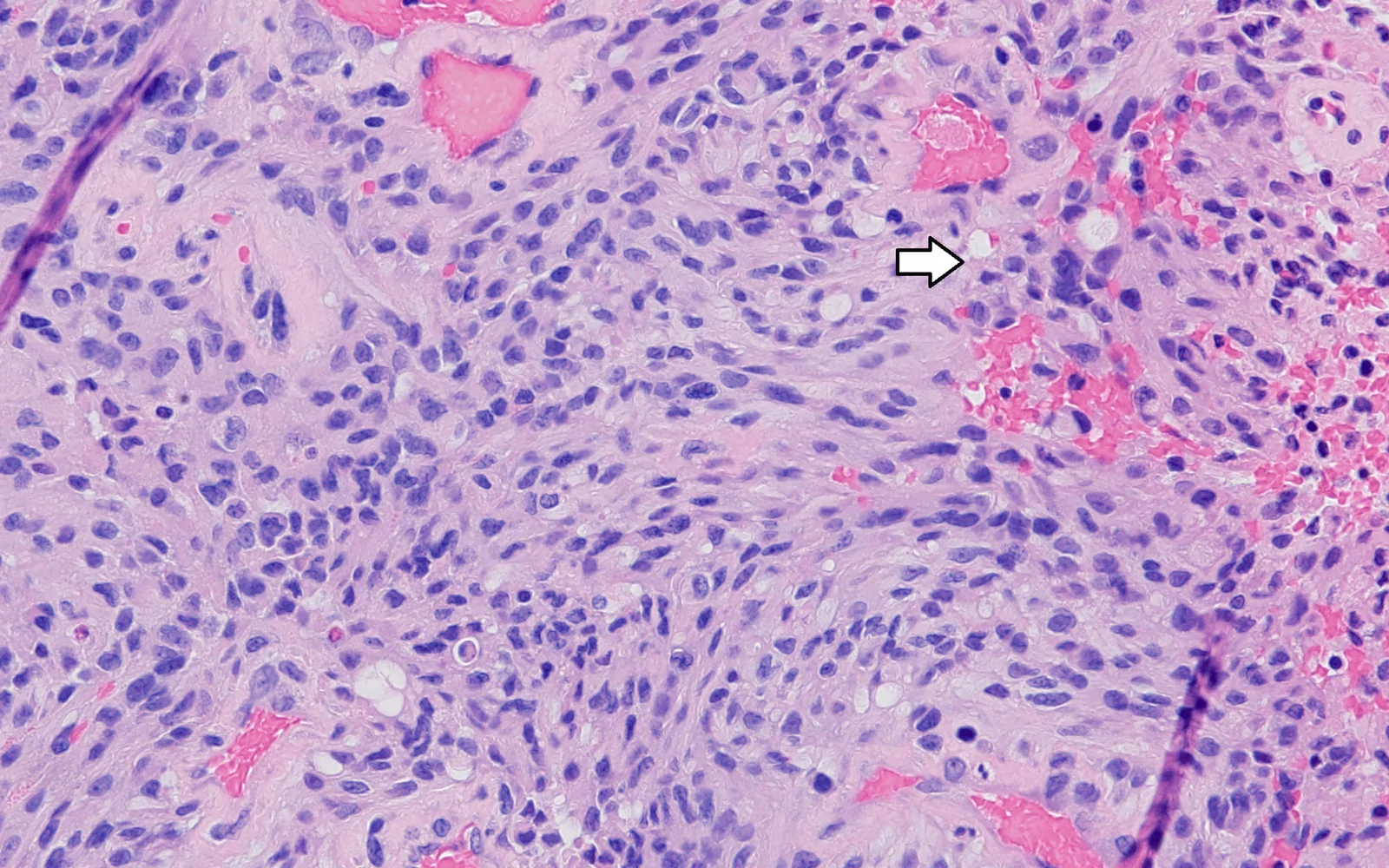
Anaplastic PXA, H&E: short fascicular arrangement of spindle cells and microvascular proliferation (indicated by arrow). PXA: pleomorphic xanthoastrocytoma; H&E: hematoxylin and eosin.

**Figure 6:**
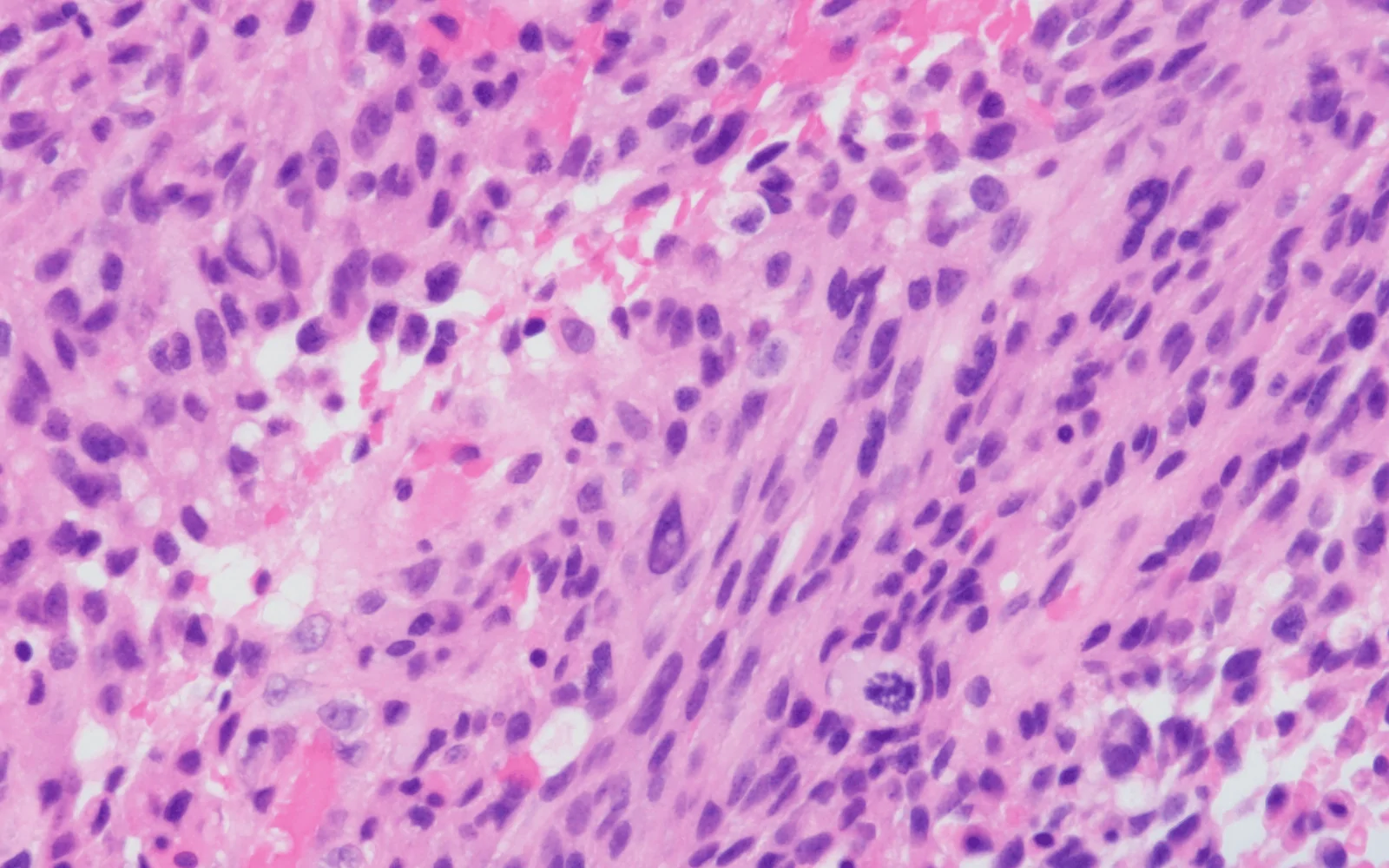
Anaplastic PXA, H&E: epithelioid morphology with highly pleomorphic cells and frequent mitoses. PXA: pleomorphic xanthoastrocytoma; H&E: hematoxylin and eosin.

Hematoxylin and eosin (H&E)-stained sections from the resected tumor revealed an expansile proliferation of predominantly spindled neoplastic astrocytes arranged in fascicles, with focal areas of pleomorphic giant cells exhibiting cytoplasmic vacuolization and xanthomatous change, characteristic of PXA. Mitotic activity was elevated at nine mitoses per 10 hpf, and scattered foci of pseudopalisading necrosis were identified, fulfilling criteria for WHO grade 3 anaplastic PXA [3]. At the tumor periphery, distinct piloid glial elements were observed, comprising elongated, bipolar piloid cells with scattered eosinophilic granular bodies and rare Rosenthal fibers (Figures 2-6), reminiscent of pilocytic astrocytoma or HGAP [6,7].

**Figure 7:**
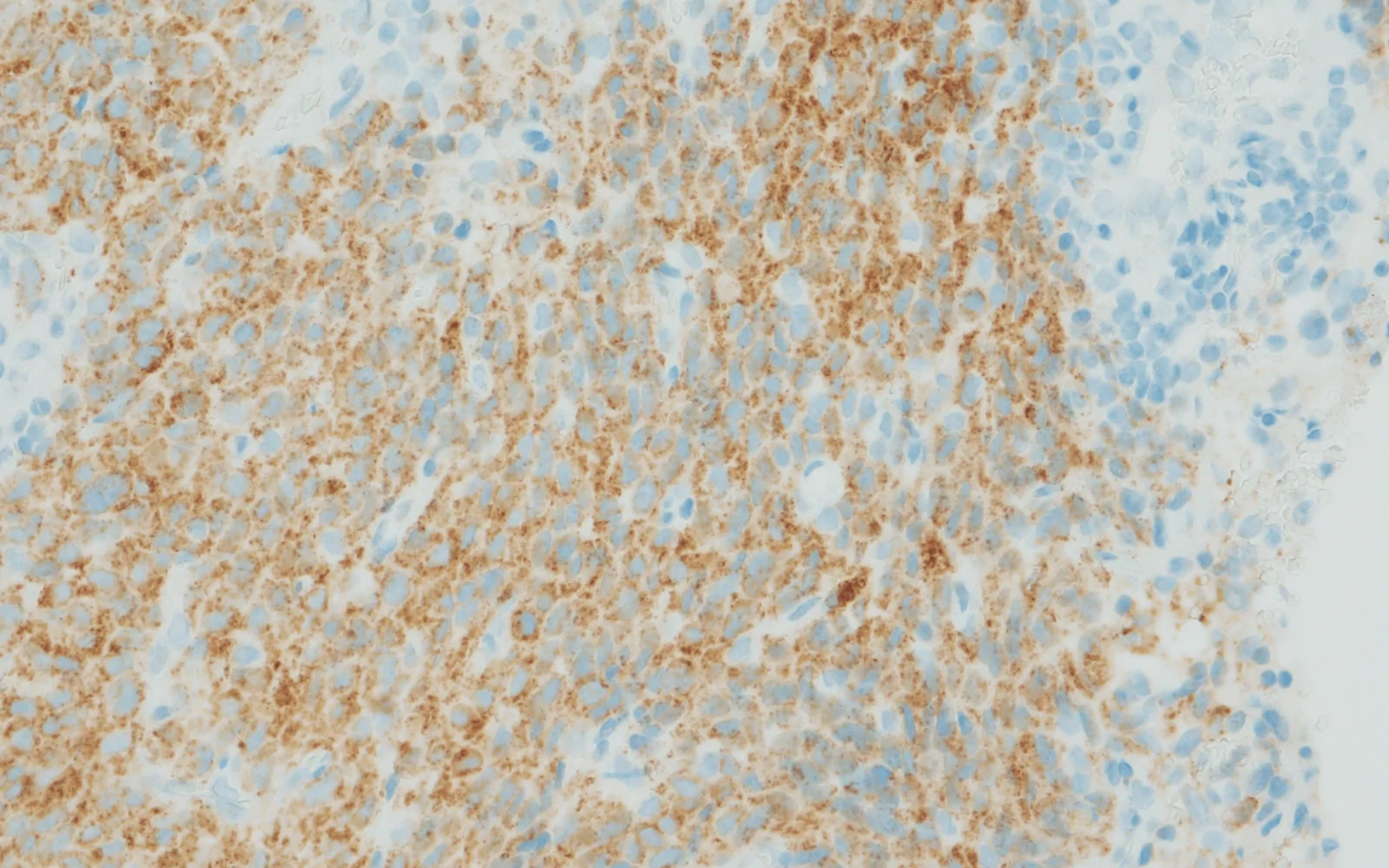
Anaplastic PXA, immunohistochemistry (IHC): strong BRAFV600E granular cytoplasmic staining. PXA: pleomorphic xanthoastrocytoma.

**Figure 8:**
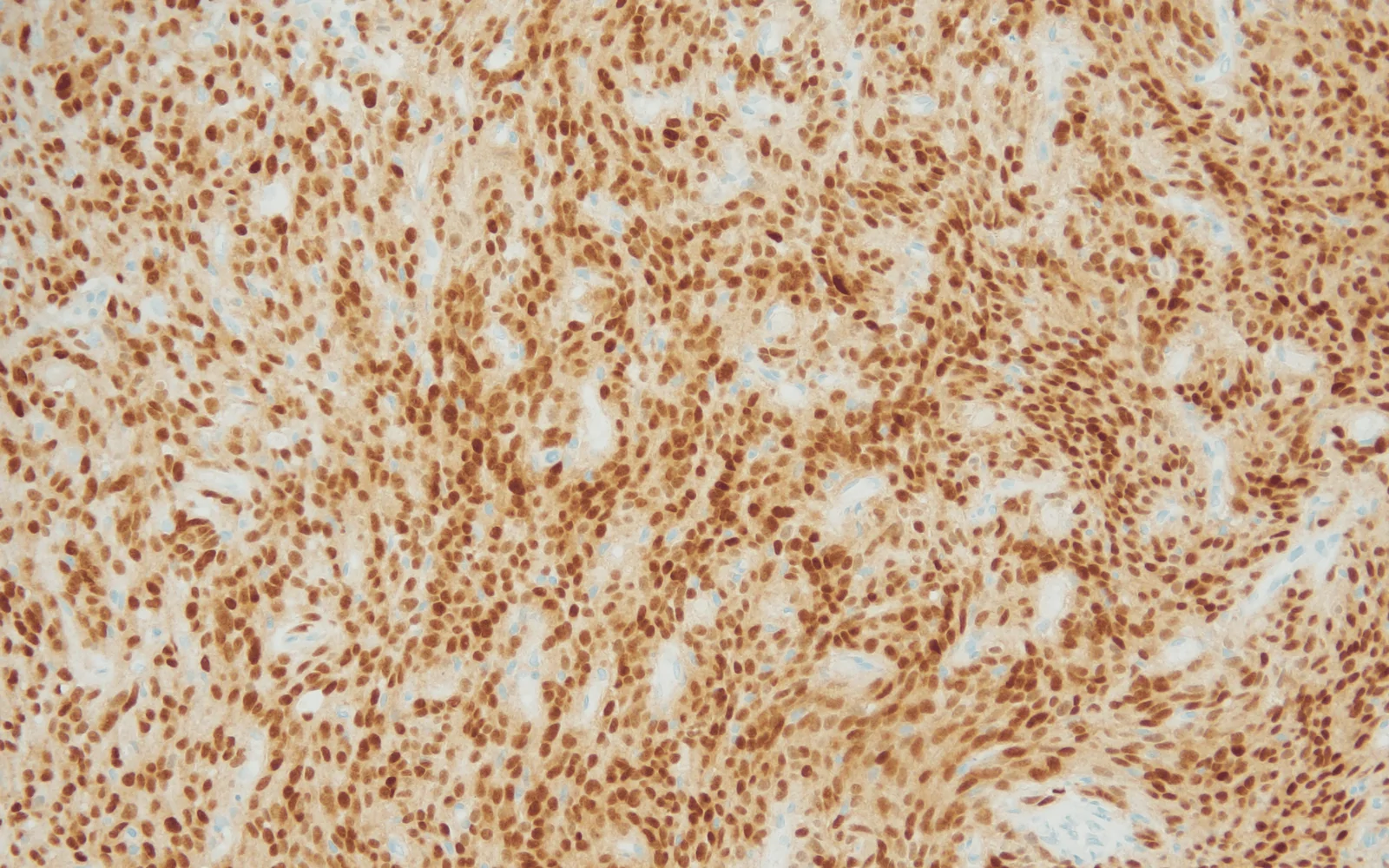
Anaplastic PXA, IHC: bright OLIG2 nuclear staining in tumor cells. PXA: pleomorphic xanthoastrocytoma; IHC: immunohistochemistry; OLIG2: oligodendrocyte lineage transcription factor 2.

Immunohistochemical studies showed diffuse positivity for glial fibrillary acidic protein (GFAP) in astrocytic components, with variable synaptophysin expression in piloid areas. Ki-67 proliferation index was elevated (15-20%) in anaplastic regions. The tumor immunohistochemistry was positive for BRAF V600E and OLIG2 (Figures 7, 8), and there was loss of INK4A/alternate reading frames (ARFs) (CDKN2A) expression.

Molecular analysis via next-generation sequencing (NGS) identified a BRAF p.V600E point mutation and an LRP1B frameshift mutation, with wild-type status for IDH1/2, TERT promoter, and ATRX. Chromosomal microarray analysis (CMA) revealed multiple copy number alterations, including whole-arm losses of chromosomes 3 and 6, homozygous deletion of 9p21.3 (encompassing CDKN2A and CDKN2B), and heterozygous losses of RB1, NF2, and phosphatase and tensin homolog (PTEN). The overall findings supported a diagnosis of pleomorphic xanthoastrocytoma (PXA), CNS WHO grade 3. However, the presence of focal piloid morphology necessitated consideration of high-grade astrocytoma with piloid features, a molecularly defined IDH-wildtype glioma with recurrent MAPK pathway alterations and frequent CDKN2A/B alterations [2,10].

## Discussion

Pleomorphic xanthoastrocytoma (PXA) is a rare WHO grade 2 tumor of the superficial cortex, predominantly affecting children and young adults, with a predilection for the temporal lobe [11]. It is characterized by large pleomorphic and multinucleated cells, spindled and lipidized (xanthomatous) cells in a fascicular pattern, eosinophilic granular bodies, and a dense reticulin network [4]. Anaplastic PXA, classified as WHO grade 3, is defined by >5/10 high-power field (hpf) mitoses and frequent necrosis, potentially arising de novo or from a pre-existing low-grade lesion [3]. The five-year overall survival for anaplastic PXA is approximately 55%, reflecting its aggressive behavior [12]. Anaplastic PXA requires differentiation from epithelioid glioblastoma (eGBM, WHO grade 4) and the recently described high-grade astrocytoma with piloid features (HGAP, WHO grade 3) (Table 1).

**Table 1:** Clinicopathological and molecular features differentiating APXA from eGBM and HGAP. GFAP: glial fibrillary acidic protein; OLIG2: oligodendrocyte lineage transcription factor 2; CD34: cluster of differentiation 34; BRAF: B-Raf proto-oncogene, serine/threonine kinase; CDKN2A/B: cyclin-dependent kinase inhibitor 2A/2B; TERT: telomerase reverse transcriptase; EGFR: epidermal growth factor receptor; MAPK: mitogen-activated protein kinase (including NF1, neurofibromin 1); ATRX: alpha-thalassemia/mental retardation syndrome X-linked; MGMT: O-6-methylguanine-DNA methyltransferase; APXA: anaplastic pleomorphic xanthoastrocytoma; eGBM: epithelioid glioblastoma; (+): gain of chromosome; (-): loss of chromosome.

Features	APXA	eGBM	HGAP
Localization	Superficial cortex (temporal most common)	Deep cortex	Posterior fossa, cerebellum
Epidemiology	Pediatric/young adult	Pediatric/adult	Adults
Peak age	26 years	55 years	40 years
Male: Female ratio	1:1	1.6:1	1:1
Radiology	Circumscribed with cystic change, leptomeninges involved	Infiltrative, necrosis + ring enhancement	Cystic with ring enhancement
Microvascular proliferation, necrosis, mitoses	Present	Present	Present
Xanthomatous cells	Present	Absent	Mostly absent
Rosenthal fibers/piloid features	Present	Absent	Present
Eosinophilic granular bodies	Present	Absent	Present
Reticulin staining	Present	Absent	Variable
Stains	GFAP+, OLIG2+, CD34+, BRAF+ (~79%)	GFAP+, OLIG2+, BRAF+ (~35%), ATRX-, IDH-	GFAP+, OLIG2+, BRAF variable, ATRX-, IDH-
Molecular profile	BRAF V600E mutation, CDKN2A/B deletion	TERT, EGFR, +7/-10	MAPK (NF1), CDKN2A, ATRX
Prognosis (five-year survival)	Better (50-57%)	Worst (6-7%)	Better (~50%)
Molecular prognostication	BRAF V600E Good, TERT Bad	MGMT Good, TERT Bad	No significant prognostic value

The presence of focal piloid features-elongated bipolar glial cells, eosinophilic granular bodies, and rare Rosenthal fibers-in this case is a striking and unusual finding. The tumor showed morphologic overlap with high-grade astrocytoma with piloid features (HGAP); however, the integrated histologic and molecular profile, particularly the presence of a BRAF p.V600E mutation and homozygous CDKN2A/B deletion, supported a diagnosis of pleomorphic xanthoastrocytoma (PXA), CNS WHO grade 3 [9]. Although piloid features are classically associated with lower-grade pilocytic astrocytomas or high-grade astrocytoma with piloid features (HGAP), their occurrence in anaplastic pleomorphic xanthoastrocytoma (PXA) is exceptionally uncommon. This rare histologic finding may have diagnostic and prognostic significance, underscoring the importance of comprehensive histopathological and molecular characterization [7,11]. Our patient, a 39-year-old male, has remained in complete remission for approximately 4.5 years post-diagnosis (as of October 2025), contrasting with the reported five-year overall survival of approximately 50-57% for pleomorphic xanthoastrocytoma (PXA), CNS WHO grade 3 [12]. The patient's durable recurrence-free survival is noteworthy despite the tumor's high-grade histologic features and is consistent with the relatively favorable clinical outcomes reported in a subset of BRAF-mutant pleomorphic xanthoastrocytoma (PXA), CNS WHO grade 3. This prolonged survival suggests that piloid features might confer a better prognosis, potentially due to a less aggressive biological behavior or enhanced responsiveness to therapy, such as the combined temozolomide and radiation regimen administered [7,13].

The molecular profile further supports this interpretation. The LRP1B mutation, though rare, may contribute to tumor progression [14]. The BRAF p.V600E mutation, present in ~80% of PXAs, offers a potential therapeutic target with inhibitors like vemurafenib [4,15], while the homozygous CDKN2A/B deletion, common in anaplastic variants, is not typically linked to grade or BRAF status but indicates a high-risk profile [2,5]. Additional losses (RB1, NF2, PTEN) align with glioblastoma-like aggressiveness, yet the absence of IDH, TERT, and ATRX mutations distinguishes this case from IDH-mutant astrocytomas [2,8]. The coexistence of piloid morphology with this molecular backdrop is unprecedented in most reported anaplastic PXAs, suggesting a hybrid entity that warrants further investigation [2]. The prognostic benefit observed here aligns with reports of prolonged survival in HGAP subsets with piloid differentiation, reinforcing the hypothesis that these features may mitigate the otherwise poor outcome of anaplastic PXA [7,13].

This case underscores the diagnostic challenge posed by overlapping features with eGBM and HGAP [1]. Although the favorable outcome observed in our patient is intriguing, the prognostic significance of focal piloid features in anaplastic PXA cannot be established from a single case, and further studies are needed to determine whether this association is reproducible. Future studies, potentially involving methylation profiling, could validate this prognostic implication and refine classification, enhancing personalized treatment strategies for such rare variants.

## Conclusions

Anaplastic pleomorphic xanthoastrocytoma (PXA) represents a highly uncommon aggressive variant that exhibits overlapping clinical, histological, immunohistochemical, and genetic characteristics with epithelioid glioblastoma and high-grade astrocytoma with piloid features, often creating substantial diagnostic difficulties. The identification of lower-grade elements, such as eosinophilic granular bodies and piloid differentiation (including Rosenthal fibers), facilitates accurate diagnosis and appears to be associated with improved long-term outcomes in affected individuals.
